# New development in CAR-T cell therapy

**DOI:** 10.1186/s13045-017-0423-1

**Published:** 2017-02-21

**Authors:** Zhenguang Wang, Zhiqiang Wu, Yang Liu, Weidong Han

**Affiliations:** 1 Molecular & Immunological Department, Bio-therapeutic Department, Chinese PLA General Hospital, No. 28 Fuxing Road, Beijing, 100853 China; 2 Department of Geriatric Hematology, Chinese PLA General Hospital, Beijing, 100853 China

**Keywords:** Chimeric antigen receptor, CAR-T, Engineered T cells, Adoptive cell therapy, Cancer treatment

## Abstract

Chimeric antigen receptor (CAR)-engineered T cells (CAR-T cells) have yielded unprecedented efficacy in B cell malignancies, most remarkably in anti-CD19 CAR-T cells for B cell acute lymphoblastic leukemia (B-ALL) with up to a 90% complete remission rate. However, tumor antigen escape has emerged as a main challenge for the long-term disease control of this promising immunotherapy in B cell malignancies. In addition, this success has encountered significant hurdles in translation to solid tumors, and the safety of the on-target/off-tumor recognition of normal tissues is one of the main reasons. In this mini-review, we characterize some of the mechanisms for antigen loss relapse and new strategies to address this issue. In addition, we discuss some novel CAR designs that are being considered to enhance the safety of CAR-T cell therapy in solid tumors.

## Background

**Fig. 1:**
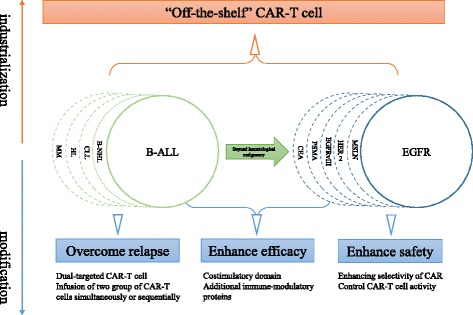
Future directions in CAR-T cell therapy. Overcoming antigen loss relapse and enhancing efficacy and safety present a principal direction of CAR-T cell therapy optimization. “Off-the-shelf” CAR-T, a biologic that is pre-prepared in advance from one or more healthy unrelated donors, validated, and cryopreserved and then can be shipped to patients worldwide, is deemed to be the ultimate product formulation. *CAR* chimeric antigen receptor, *CAR-T cell* chimeric antigen receptor-modified T cell, *B-ALL* B cell acute lymphoblastic leukemia, *B-NHL* B cell non-Hodgkin’s lymphoma, *CLL* chronic lymphocytic leukemia, *HL* Hodgkin’s lymphoma, *MM* multiple myeloma, *EGFR* epidermal growth factor receptor, *MSLN* mesothelin, *HER2* human epidermal growth factor receptor-2, *EGFRvIII* variant III of the epidermal growth factor receptor, *PSMA* prostate-specific membrane antigen, *CEA* carcinoembryonic antigen

Chimeric antigen receptor (CAR) is a modular fusion protein comprising extracellular target binding domain usually derived from the single-chain variable fragment (scFv) of antibody, spacer domain, transmembrane domain, and intracellular signaling domain containing CD3z linked with zero or one or two costimulatory molecules such as CD28, CD137, and CD134 [1–3]. T cells engineered to express CAR by gene transfer technology are capable of specifically recognizing their target antigen through the scFv binding domain, resulting in T cell activation in a major histocompatibility complex (MHC)-independent manner [4]. In the past several years, clinical trials from several institutions to evaluate CAR-modified T cell (CAR-T cell) therapy for B cell malignancies including B cell acute lymphoblastic leukemia (B-ALL), B cell non-Hodgkin’s lymphoma (B-NHL), chronic lymphocytic leukemia (CLL), and Hodgkin’s lymphoma (HL) have demonstrated promising outcomes by targeting CD19 [5–13], CD20 [14], or CD30 [15], where mostly compelling success has been achieved in CD19-specific CAR-T cells for B-ALL with similar high complete remission (CR) rates of 70~94% [5–8, 12]. This significant efficacy not only leads to an impending paradigm shift in the treatment of B cell malignancies but also results in a strong push toward expanding the uses of CAR-T cell therapy for solid tumors. However, the preliminary outcomes of clinical trials testing epidermal growth factor receptor (EGFR) [16], mesothelin (MSLN) [17, 18], variant III of the epidermal growth factor receptor (EGFRvIII) [19], human epidermal growth factor receptor-2 (HER2) [20, 21], carcinoembryonic antigen (CEA) [22], and prostate-specific membrane antigen (PSMA) [23] in solid tumors are less encouraging. Moreover, rapid death caused by the off-tumor cross-reaction of CAR-T cells has been reported [20], highlighting the important priority of enhancing CAR-T cell therapy safety. Overall, there remain several powerful challenges to the broad application of CAR-T cell therapy in the future: (1) antigen loss relapse, an emerging threat to CAR-T cell therapy, mainly observed in anti-CD19 CAR-T cells for B-ALL; (2) on-target/off-tumor toxicity resulting from the recognition of healthy tissues by CAR-T cells which can cause severe and even life-threatening toxicities, especially in the setting of solid tumors; (3) there is less efficacy in solid tumors, mainly due to the hostile tumor microenvironment; (4) difficulty of industrialization because of the personalized autologous T cell manufacturing and widely “distributed” approach. How to surmount these hurdles presents a principal direction of CAR-T cell therapy development, and a variety of strategies are now being investigated (Fig. 1). Here, we mainly focus on the new CAR design to address tumor antigen escape relapse and to enhance the safety of CAR-T cells in solid tumors.

### How to overcome antigen loss relapse in hematological malignancies

Antigen escape rendering CAR-T cells ineffective against tumor cells is an emerging threat to CAR-T cell therapy, which has been mainly seen in the clinical trials involving CD19 in hematological malignancies. It appears to be most common in B-ALL and has been observed in approximately 14% of pediatric and adult responders across institutions (Table 1) [5, 24–26]. It has also been documented in CLL [27, 28] and primary mediastinal large B cell lymphoma (PMLBCL) [29]. Indeed, it has also been noted in patients who received blinatumomab [30], a first-in-class bispecific T engager (BiTE) antibody against CD19/CD3 [31, 32], which has also shown promising efficacy in B cell malignancies [33–35], implying that this specific escape may result from the selective pressure of CD19-directed T cell immunotherapy [36]. Moreover, tumor editing resulting from the selective pressure exerted by CAR-T cell therapy also can be seen when beyond CD19; we observed that a patient with acute myeloid leukemia (AML) experienced selected proliferation of leukemic cells with low saturation of CD33 expression under the persistent stress of CD33-directed CAR-T cells [37]. Actually, antigen escape has also been reported in the experimental study of solid tumor, where targeting HER2 in a glioblastoma cell line results in the emergence of HER2-null tumor cells that maintain the expression of non-targeted, tumor-associated antigens [38]. These findings suggest that treatment of patients with specifically targeted therapies such as CAR-T cell therapy always carry the risk of tumor editing, highlighting that development of approaches to preventing and treating antigen loss escapes would therefore represent a vertical advance in the field.

**Table 1:** Summary of reported CD19-negative relapse in trials of anti-CD19 CAR-T cells for B-ALL

Treating institute	Patient populations	Construct (scFv-Hinge-TM-CD-SD)	Gene transfer method	Conditioning therapy	Infused cell dose	Responses observed	Reported relapse
MSKCC [26]	Adult 33 32 evaluable for response	SJ25C1-CD28-CD3ζ	Retrovirus	Cy or Cy/Flu	1–3 × 10^6^ CAR^+^ T cells/kg	CR: 29/32 (91%)	14 relapse with 2 CD19− relapse
Upenn [24]	Pediatric and young adult 59	FMC63-CD8α-4-1BB-CD3ζ	Lentivirus	Investigator’s choice	10^7^–10^8^ cells/kg with a transduction efficiency of 2.3–45%	CR: 55/59 (93%)	20 relapse with 13 CD19− relapse
NCI [25]	Young adult 38	FMC63-CD28-CD3ζ	Retrovirus	Cy/Flu or FLAG or IE	1 or 3 × 10^6^ CAR^+^ T cells/kg	CR: 23/38 (61%)	2 CD19− relapse
FHCRC [5]	Adult 30 29 evaluable for response	FMC63-IgG4 CD28-4-1BB-CD3ζ	Lentivirus	Cy ± etoposide or Cy/Flu	2 × 10^5^ or 2 × 10^6^ or 2 × 10^7^ CAR^+^ T cells/kg (1:1 CD4+:CD8+)	CR: 27/29 (93%)	9 relapse with 2 CD19− relapse

*MSKCC* Memorial Sloan Kettering Cancer Center, *Upenn* University of Pennsylvania, *NCI* US National Cancer Institute, *FHCRC* Fred Hutchinson Cancer Research Center, *scFv* single-chain variable fragment, *B-ALL* B cell acute lymphoblastic leukemia, *Cy* cyclophosphamide, *Flu* fludarabine, *FIAG* fludarabine + Ara-c + G-CSF, *IE* ifosfamide/etoposide, *CR* complete remission, *CAR-T* chimeric antigen receptor-modified T cell

Given the extensive trials to date involving CD19, we have gained a much better understanding regarding possible mechanism of these phenomena. Although all these antigen escape relapses are characterized by the loss of detectable CD19 on the surface of tumor cells, multiple mechanisms are involved. One mechanism is that CD19 is still present but cannot be detected and recognized by anti-CD19 CAR-T cells as its cell surface fragment containing cognate epitope is absent because of deleterious mutation and alternative splicing. Sotillo and colleagues showed a CD19 isoform that skipped exon 2 (Δex2) characterized by the loss of the cognate CD19 epitope necessary for anti-CD19 CAR-T cells is strongly enriched compared to prior anti-CD19 CAR-T cell treatment in some patients with B-ALL who relapse after anti-CD19 CAR-T cell infusion. They estimated that this type of antigen escape relapse would occur in 10 to 20% of pediatric B-ALL treated with CD19-directed immunotherapy. Moreover, they found that this truncated isoform was more stable than full-length CD19 and partly rescued defects in cell proliferation and pre-B cell receptor (pre-BCR) signaling associated with CD19 loss [39]. Similar to that observed in B-ALL, a biopsy of renal lesion from a patient with persistent renal involvement by PMLBCL 2 months after anti-CD19 CAR-T cell infusion indicated that activated anti-CD19 CAR-T cells could infiltrate the tumor; however, the PMLBCL clone is absent on surface CD19 but shows positive cytoplasmic expression [29]. These findings imply that it may make sense to simultaneously evaluate the cytoplasmic and membranous expression of CD19 by flow cytometry and immunohistochemistry. Moreover, leukemic lineage switch provides new insights into mechanisms of immune escape from targeted immunotherapy [40]. Gardner et al. reported on 2 of 7 patients with B-ALL harboring rearrangement of the mixed lineage leukemia (MLL) gene and achieving molecular CR after anti-CD19 CAR-T cell infusion developing AML that was clonally related to their B-ALL within 1 month after anti-CD19 CAR-T cell infusion [41]. Both aforementioned phenomena can be recapitulated in a syngeneic murine model where mice bearing E2a:PBX1 leukemia are treated with murine anti-CD19 CAR-T cells [42]. Intriguingly, researchers demonstrated that earlier relapses maintained pre-B phenotype with isolated CD19 loss, whereas later relapses involved multiple phenotypic changes, including the loss of additional B cell markers. Moreover, B cell-associated transcripts and an increase in the expression of myeloid or T cell genes consistent with lineage switching were also confirmed in later relapses by unsupervised clustering of RNA sequencing, implying that lineage switching results from reprogramming rather than depletion of CD19 alone. Outgrowth of preexisting rare CD19-negative malignant cells as a consequence of immunoediting also can lead to B-ALL cells escape anti-CD19 CAR-T cells killing, which is described by Ruella et al. in research focusing on dual CD19 and CD123 CAR-T cells [43]. They showed the existence of rare CD19-negative CD123-positive cells at baseline in the samples from patients with B-ALL. These cells emerged after anti-CD19 CAR-T cell administration, which accounts for the CD19-negative relapse as CD19-CD123+ blasts carried the disease-associated genetic aberration and can lead to the reconstitution of the original B-ALL phenotype when those cells are injected into NOD/SCID/gamma (NSG)-chain-deficient mice. On this basis, researchers developed a dual CAR-expressing construct that combined CD19- and CD123-mediated T cell activation and proved that this dual antigen receptor can treat and prevent CD19-loss relapses in a clinically relevant preclinical model of CD19-negative leukemia escape. Similar phenomena have also been shown in CLL, in which CD19-negative escape variants were selected due to the treatment pressure exerted by anti-CD19 CAR-T cells, which also resulted in the transformation from CLL to plasmablastic lymphoma [28].

Novel strategies to offset tumor antigen loss relapse are mainly geared toward generating T cells capable of recognizing multiple antigens, in which dual-targeted CAR-T cells have been actively investigated in preclinical research and have two main patterns: modifying individual T cells with two distinct CAR molecules with two different binding domains (known as dual-signaling CAR) [38, 43] or with one CAR molecule containing two different binding domains in tandem (termed TanCAR) [44–46]. The prerequisite of the dual-targeted CAR, either dual-signaling CAR or TanCAR, is that either antigen input can trigger robust anti-tumor activity, which ensures that there is always another antigen input that can work well and control antigen loss relapse in the setting of one antigen escape. The concept is simple but is still a challenge in the context of limited choices of clinically validated antigens and the constraint of suitable epitope selection in the setting of TanCAR [47]. Besides CD19, other pan-B cell markers such as CD20 [14] and CD22 [36] can be proposed as a target for dual-targeted CAR in B cell malignancies as these antigen-directed CARs have been tested in humans and presented encouraging outcomes in early clinical trials. Moreover, CD123 (also called IL-3 receptor α chain) is also an ideal option for the target selection of dual-targeted CAR [43, 48]. It is worth noting that enhanced anti-tumor activity was demonstrated by dual-signaling CAR or TanCAR compared to the unispecific CAR or pooling unispecific CAR when both antigens are expressed on the tumor cell surface [43, 45], highlighting the safety concern. This design potentially increases the risk of CRS and on-target/off-tumor recognition resulting from more significant CAR-T cell expansion in vivo and cytokine release. In addition, whether the enhanced immune pressure directly caused by the enhanced anti-tumor activity can lead to loss of both antigens simultaneously because of tumor adaptation is another concern; hence, targeting two antigens may not be enough, and more studies are needed to determine the optimal antigen combination for each cancer. Other tactics to achieve dual recognition are pooling unispecific CAR-T cells; however, coadministering two CAR-T cell populations may result in the disproportionate expansion of one CAR-T cell suggested by the observation that anti-CD19 CAR-T cells have a significant growth advantage over CD20-specific CAR-T cells when in a coculture system, leading to a net decline in CD20-specific CAR-T cell count despite the presence of CD20 antigen [44]. Furthermore, sequentially infusing two groups of CAR-T cells [49] is also an alternative to avoid antigen escape and could circumvent the disproportionate expansion as seen in pooling CAR-T cells. However, it still is a combination of two groups of CAR-T cells as pooling CAR-T cells, resulting in a relatively long clinical time frame. Taken together, we would prefer dual-targeted CAR-T cells, but much additional work is needed to test and optimize this strategy before it can be translated into humans. Right now, our group are testing CD19/CD20 and CD19/CD22 dual-targeted CARs for B cell malignancies in experimental studies. Moreover, based on the lessons learned from the patient who received anti-CD33 CAR-T cells [37], a CD33/CD123 dual-targeted CAR for AML has already been included in our development pipeline.

On the other hand, selective targeting of cancer stem cells (CSCs) rather than tumor cells for CAR-T cell therapy may lead to better cancer treatment [50]. The reason for that is CSCs retain extensive self-renewal and tumorigenic potential, determining a tumor’s behavior, including proliferation and progression [51]. CD133 is an attractive therapeutic target for CAR-T cell therapy when targeting CSCs [52]. We first tested a CD133-directed CAR characterized by a shorted promoter in an effort to minimize the risk of on-target/off-tumor recognition in humans. A patient with cholangiocarcinoma, who progressed after anti-EGFR CAR-T cell therapy, in turn had another partial response with severe but can be managed epidermal/endothelial toxicities may due to the cross-reaction with CD133 expressed on normal epithelium and vascular endothelium after treated with CD133-directed CAR. These findings provide the proof-of-concept evidence that anti-CD133 CAR confers effective anti-tumor immunity which may contribute to the long-term disease control, but the on-target/off-tumor toxicity warrants further evaluation.

At the same time, some attention should be paid to the endogenous immune system, albeit it cannot be effective against tumor cells because of a lack of sufficient tumor-specific T cells as well as suppression by the tumor immunosuppressive microenvironment. By increasing cytokine production (e.g., IL-12) or the addition of immune checkpoint inhibitors (e.g., anti-PD-1/PD-L1/CTLA-4 monoclonal antibodies), existing endogenous anti-tumor immune cells can be rescued and may even induce epitope spreading [53]. Epitope spreading is a process in which antigenic epitopes distinct from and non-cross-reactive with an inducing epitope become additional targets of an ongoing immune response [54], which provides the rationale for recruitment of endogenous immune cells to recognize and eradicate a new relapsed tumor clone. However, this hypothesis needs to be further verified in upcoming clinical trials. The most thorough reconstitution of the immune system is allogeneic stem cell transplantation (allo-SCT), in which a patient’s own hematopoiesis is ablated through high-dose chemotherapy or radiation. Regenerated normal hematopoiesis including a new immune system can potentially recognize and destroy either type of tumor antigen escape relapse clone [36]. Significantly, allo-SCT is performed at several institutions for patients with B-ALL achieving CR after CAR-T cell therapy, and it demonstrated reduced relapse rate [25]. However, the Memorial Sloan Kettering Cancer Center (MSKCC) group showed that among the 36 patients in CR following CAR-T cell infusion, 6-month overall survival (OS) did not differ significantly between patients who underwent allo-SCT (70%) and those who did not (64%) [55]. We suggest pursuit of consolidative allo-SCT for patients with B-ALL who achieve CR after CAR-T cell therapy regardless of the persistence of CAR-T cells in vivo, especially for patients who are thought to be at higher risk of relapse.

### How to enhance safety of CAR-T cells in solid tumors

Severe treatment-related toxicities mainly due to the on-target/off-tumor recognition are another obstacle for CAR-T cell therapy beyond hematological malignancies [20]. How to abrogate the toxicity is crucial for this emerging technology and has become a research hotspot. Strategies for enhancing the safety of CAR-T cell therapy in solid tumors fall into several categories (Table 2).

**Table 2:** Strategies for enhancing safety of CAR-T cells in solid tumors

Strategy			Phase	Reference
Enhancing selectivity of CAR	Selecting safer antigen	Tumor-specific antigen	Clinical trial	[19]
		Aberrantly glycosylated antigens	Preclinical research	[57]
		TCR-like CAR	Preclinical research	[60–62]
	Combinatorial antigen targeting	Complementary signaling	Preclinical research	[64, 65]
		SynNotch/CAR circulation	Preclinical research	[68]
		iCAR	Preclinical research	[70]
	Turning sensitivity of scFv	Turning the affinity	Preclinical research	[74, 75]
	Masked CAR		Preclinical research	[78]
Control CAR-T cell activity	Limiting CAR expression	Transient mRNA CAR	Clinical trial	[17, 18]
	Switchable CAR-T cell	Dimerizing small molecules	Preclinical research	[84, 85]
		Tumor targeting antibody	Preclinical research	[86, 88, 90]
	Suicide gene	iCasp9	Clinical trial	[92]
		Antibody-mediated depletion	Clinical trial	[5, 9]

*CAR* chimeric antigen receptor, *CAR-T cell* chimeric antigen receptor-modified T cell, *TCR* T cell receptor, *scFv* single-chain variable fragment, *SynNotch* synthetic Notch receptors, *iCAR* inhibitory chimeric antigen receptor, *iCasp9* inducible caspase-9

### Enhancing selectivity of CAR

#### Selecting safer antigen

CAR can only attack cells expressing targeted antigen; hence, the most direct and effective means to surmount off-tumor toxicities while not compromising efficacy is by targeting truly tumor-specific antigen expressed only on the tumor cells. However, the vast majority of CAR targets have been tumor-associated antigens (TAAs) that are overexpressed on tumor cells but also shared by normal “bystander” cells. Thus far, the only truly tumor-specific antigen for CAR is EGFRvIII, which is strictly confined to human cancer (most frequently observed in glioblastoma) [56]. An early outcome of EGFRvIII-specific CAR in 9 patients with EGFRvIII-positive glioblastoma demonstrated that the infusion was well-tolerated without off-tumor toxicities [19].

Of note, Posey et al. demonstrated that aberrantly glycosylated antigen-Tn-MUC1 can also be proposed as an ideal target for CAR-T cell therapy as selective recognition of Tn- and STn-positive malignant tumors has been achieved by T cells expressing 5E5 CAR, a newly designed CAR containing scFv derived from antibody 5E5 specific for Tn and STn glycoepitopes [57]. Moreover, robust cytotoxicity of 5E5 CAR-T cells in murine models of cancers as diverse as leukemia and pancreatic cancer also have been observed. Although much remains to be learned, these findings provide the proof-of-concept evidence that aberrantly glycosylated antigens can be proposed as a safer alternative than TAA for CAR-T cell therapy.

If we turn our attention from membrane surface molecules to the intracellular and/or secreted molecules, target selection becomes rich in diversity. Cancer/testis antigens (e.g., NY-ESO-1 and MAGE-A3) or differentiation antigens (e.g., gp100 and MART1) represent the most attractive targets for immunotherapy since these antigens are expressed only by tumor cells and spermatogenic cells from the testis or in a lineage-restricted manner [58]. However, antigens recognized by natural T cell receptor (TCR) through peptides/MHC engagement are invisible to conventional CAR as it can only recognize the membrane surface antigen. One intriguing strategy for expanding the antigenic repertoire to those antigens is using TCR-like antibody, an antibody directed to peptide-MHC (pMHC) complexes that can mimic the fine specificity of tumor recognition by TCR while having higher affinity than that of TCR [59]. T cells engineered to express the CAR comprising scFv derived from TCR-like antibody such as PR1/human leukocyte antigen (HLA-A2) or PR1/HLA-A2 alpha-fetoprotein (AFP)/HLA-A*02:01, gp100/HLA-A2 have been tested in vitro and in vivo [60–62], and preliminary results demonstrate that this design is feasible. However, several limitations are worth noting: First, TCR-like CAR is HLA restricted; thus, the activation of TCR-like CAR-T cells is not MHC independent. Second, potential off-target/off-tumor toxicity results from the cross-reactivity of these receptors with nonidentical yet sequence-related HLA-I-binding peptides presented by vital cells. Third, the extent of affinity constraints for each peptide/MHC complexes is unclear; elegant optimization is needed [63].

#### Combinatorial antigen targeting

Highly specific targets for CAR-T cell therapy are very less; for a large majority of TAAs, one strategy for enhancing the specificity of CAR is combinatorial antigen (mainly dual antigen) rather than one antigen targeting, endowing CAR-T cells with the ability to discriminate between target and off-target cells.

One design of combinatorial antigen targeting is simultaneously co-expressing two receptors with different binding domain in the same T cell population. Of the two receptors, one is a CAR containing CD3z signaling domain alone and specific for one antigen, which can provide the T cell activation signaling function. Another receptor is a chimeric costimulatory receptor (CCR) that recognizes another antigen, providing the costimulation signaling function by CD28 and/or CD137. Theoretically, the T cells engineered with these complementary dual receptors can only be fully activated in the context of the presence of both antigens. In a proof-of-concept experiment, Wilkie et al. showed that the T cells transduced with a CAR specific for HER2 and a CCR specific for MUC1 elicited enhanced T cell proliferation, which is dependent on the engagement of HER2 and MUC1. However, the cytolytic activity of these T cells is only dependent on the engagement of HER2 irrespective of MUC1, which was also observed [64], thus challenging the implementation of these receptors. This non-double-positive tumor-limiting T cell reactivity also resulted in the failure of Kloss’ early experiments focusing on dual-targeted T cells (CD19 and PSMA) [65]. To remedy this failure, Kloss et al. constructed three anti-prostate stem cell antigen (PSCA) CARs with different binding affinity for PSCA by combination with the same CCR specific for PSMA. The author tested these receptors in a human xenograft tumor model in immunodeficient mice bearing tumors expressing PSCA and/or PSMA. Significantly, only the T cells expressing CAR with lower binding affinity for PSCA demonstrated reactivity strictly specific for PSCA and PSMA double-positive tumor cells, providing an alternative option for increasing the CAR specificity. However, practical questions remain to be investigated, such as suitable TAA pairs uniquely expressed on tumor cells with the desired range of affinity selection [66].

Another design of combinatorial antigen targeting is taking advantage of the synthetic Notch receptors (synNotch), a new class of modular receptors comprising extracellular recognition domain; the transmembrane “core” domain; and the intracellular transcription domain that can be cleaved and released by a transcriptional activation domain translocating to the nucleus and regulating transcription upon ligand engagement [67]. By introducing the synNotch platform, Roybal et al. constructed two combinatorial antigen recognition T cell circuits [CD19 synNotch/MSLN CAR, green fluorescent protein (GFP) synNotch/CD19 CAR] and demonstrated that these receptors could conditionally express CARs specific for a second antigen in the presence of the first antigen-specific for the synNotch receptor [68]. Furthermore, in Jurkat T cells expressing CD19/MSLN, the author observed that the effective half-time for occurring CAR expression, T cell activation, and CAR expression decay without synNotch stimulus were ~6, ~7, and ~8 h, respectively; this implies that these T cells encounter the first antigen in one healthy cell, and soon after the recognition of the second antigen in a different healthy cell, they can only be transiently activated when the CAR expression was downregulated because of the absence of the first antigen. The author further tested GFP and CD19 dual-targeted human primary CD4+ and CD8+ T cells in a human xenograft two-tumor model using K562 as a target; they observed the selective clearance of CD19+GFP+ tumors rather than CD19+GFP− “bystander” tumors (serving as surrogate “healthy tissue”) in the same mice. Together, these findings not only underscored the initial success of the synNotch/CAR system in enhancing the specificity of CAR-T cell therapy but also suggested that this system can potentially expand to a wider range of tumors. However, the potential toxicity toward normal human tissue, especially in the event of a second antigen presence, is still a concern as the abovementioned transient activation of T cells. Moreover, CD19 and MSLN studied in this experiment are actually not co-expressed in one tumor cell. Together with immunogenicity concerns arising from the use of multiple non-human transcriptional regulators (Gal4, tTA), much additional work is required before these types of T cell can be tested clinically [69].

Instead, if the dual antigens are simultaneously expressed on healthy cells rather than on tumor cells, the combination of inhibitory receptors (known as iCAR) specific for the antigen present on normal but not on tumor cells will protect the normal cells from a CAR-T cell-mediated attack because of negative signaling conferred by iCAR. Fedorov et al. pioneered an anti-PSMA iCAR carrying intracellular tails of CTLA-4 or PD-1 and tested whether these receptors have the ability to block TCR- or CAR-driven T cell functionality in vitro and in vivo [70]. This proof-of-concept experiment demonstrated that the iCAR can inhibit the response mediated by either TCR or CAR in an antigen-restricted manner. Moreover, this inhibition mediated by iCAR is in a temporary and reversible manner suggested by sequential T cell stimulation by target and off-target cell experiments, which ensure that most of the T cells’ previous engagement of iCAR can retain the functionality, albeit a small part of those T cells may be anergized over time. In an in vitro coculture system mixing GFP+CD19+ target AAPCs and mCherry+CD19+PSMA+ off-target AAPCs at a 1:1 ratio, T cells expressing the PD-1 iCAR and anti-CD19 CAR containing CD28 and CD3z signaling domain showed preferential elimination of the target cells while sparing the off-target cells. Together with the consistent results observed in NSG mice bearing a mixture of NALM/6 and NALM/6-PSMA tumor cells indicates those T cells can selectively protect off-target cells without abrogating rejection of the target cells in vitro and in vivo. This strategy is practically attractive for the antigen broadly expressed in normal human tissue but downregulated on tumor cells such as cell surface tumor suppressor antigens and HLA molecules, which may be targeted by iCAR to protect graft-versus-host disease (GVHD) target tissues without impairing graft versus tumor (GVT) in the setting of donor lymphocyte infusion (DLI). However, for each targeted antigen, iCAR needs elegant modification in scFv affinity, receptor expression level, and CAR/iCAR ratio as all these factors are crucial for iCAR functionality.

#### Tuning the sensitivity of CAR

It is well recognized that there is a TCR affinity window in which TCR with higher affinity can improve the recognition of the target antigen. However, beyond the TCR affinity threshold for maximal T cell anti-tumor activity, T cell activation cannot be improved or even be attenuated by further enhancement; furthermore, the risk of cross-reactivity with other self-derived pMHC complex may increase [71, 72]. Similar phenomena were also observed in the context of CAR, in which T cell activation is mediated by the antibody-derived scFv recognition of the target antigen [73]. Recently, two studies further demonstrated that by turning the affinity of a CAR, CAR-T cells could discriminate between tumor cells and normal cells that express lower or normal levels of the same antigen while retaining potent efficacy in vivo [74, 75]. Turning sensitivity of CAR by scFv affinity provides an alternative approach to empowering wider use of those targets overexpressed on tumor cells for CAR-T cell therapy. However, the optimal affinity for a scFv in the CAR format also depends on the location of the target epitope, antigen density, length of spacer, and other parameters such as the CAR expression level and the nature of the signaling domain; thus, case-by-case testing is necessary for an optimal CAR design [76].

#### Masked CAR

Protease-activated antibody (pro-antibody) is an antibody characterized by antigen-binding sites that are masked until the antibody is activated by proteases commonly found in the tumor microenvironment [77]. Desnoyers et al. designed an EGFR-targeting pro-antibody (PB1) on the basis of cetuximab, and demonstrated that PB1 was relatively inert in healthy non-human primates, but could be locally activated and showed comparable efficacy to cetuximab in two mouse models at clinically accessible drug exposures [78]. Moreover, a higher protease activity rate was observed in a collection of human tumor samples from lung and colon cancer patients, suggesting that most of EGFR-positive human tumors have the potential to activate PB1. Significantly, PB1 alleviates the dose-limiting cutaneous toxicity compared to that caused by cetuximab in female cynomolgus monkeys, implying that the PB1 could be stably masked and inactive in healthy tissues. Thus, these findings suggest that using the scFv derived from those pro-antibody represents an attractive strategy for enhancing the selectivity of CAR toward targets shared with healthy tissues [79]. However, the underlying mechanisms of activation remains unclear, and more clinical models are needed to further determine the safety before testing in clinical trials.

### Control CAR-T cell activity

#### Limiting CAR expression

Presently, the most common gene transfer strategies for clinical work are viral techniques such as the retrovirus or lentivirus that can result in permanent transgene encoding CAR expression; however, these are disadvantageous when severe toxicity related to CAR-T cell therapy occurs [80]. One of the non-viral approaches, electroporation of CAR mRNA characterized by transient CAR gene expression, is regarded as potentially safer than the viral techniques when introducing a novel CAR into patients [81]. Investigators at the University of Pennsylvania (Upenn) first evaluated the MSLN-specific mRNA CAR-T cells in patients with MSLN-expressing solid tumors (NCT01355965) on the basis of the encouraging results of preclinical studies [81, 82] and demonstrated the feasibility and safety of this novel strategy. Together with the anti-tumor activity observed, this supported the development of the mRNA CAR-based strategies for solid tumors [17, 18]. It is worth noting that multiple infusions are necessary for mRNA CAR-T cells due to the transient expression of transgene, enhancing the risk of anaphylaxis as reported [18]. Taken together, anti-MSLN CAR-T cells transduced with lentivirus were designed and tested in the subsequent clinical trials based on the safety profile shown in the MSLN-specific mRNA CAR-T cells [83].

#### Switchable CAR-T

The switchable CAR is a novel design characterized by incorporating switch molecules comprising dimerizing small molecules or a tumor targeting antibody as a bridge to link the two adjacent domains of the CAR structure [84, 85] or tumor antigen and CAR-T cells [86–89], by which the anti-tumor activity of the CAR-T cells is strictly dependent on the receptor complex formation in the presence of those switch molecules, opening up opportunities to remotely control or terminate the CAR-T cell response to avoid off-target toxicity that can occur immediately after T cell infusion. Wu et al. showed a switchable CAR design, whereby separate extracellular antigen-binding domain and intracellular signaling component can be assembled through an FKBP-FRB module only in the presence of heterodimerizing small molecules (rapamycin analog AP21967) confirmed by single-molecule imaging [85]. Wu et al. also observed the efficient killing of target cells by switchable CAR-T cells in vitro and in vivo, and this response was regulated in a titratable manner. Similar outcomes were observed in another switchable CAR by using a system that is directly integrated into the hinge domain that separates the scFv from the cell membrane [84]. Alternatively, a group at the California Institute for Biomedical Research developed antibody-based switches with site-specific incorporation of fluorescein isothiocyanate (FITC) or peptide neo-epitope (PNE) into a tumor antigen-specific antibody, which can redirect the CAR-T cells specific for corresponding FITC or PNE to tumor cells expressing the same tumor antigens and forming a switch-dependent immunological synapse [86, 88, 90]. They tested this system in B cell malignancies and breast tumors by targeting CD19, CD22, and HER2 and demonstrated that these switchable CAR-T cells have potent antigen-specific and dose-dependent anti-tumor activity, providing an attractive way to improve the safety of CAR-T cell therapy in the clinic and suggesting that these switchable CAR-T cells could be applicable to a wide range of tumor antigens.

#### Suicide gene

Unlike the above-described that the CAR-T cell response can be turned on again when the heterodimerizing small molecules are present, the depletion of CAR-T cells by incorporation of a suicide gene such as inducible caspase-9 (iCasp9) enzyme is irreversible [91]. Di Stasi et al. first tested the iCasp9-modified donor T cells in haploidentical SCT recipients and showed that more than 90% of the modified T cells were depleted within 30 min after administration of a single dose of dimerizing agent AP1903 among 4 patients developing GVHD [92]. This rapid onset of action resulted in the fast (within 24 h) and permanent abrogation of GVHD, albeit there remained a small number of residual iCasp9-modified T cells. Currently, several clinical trials evaluating iCasp9-modified CAR-T cells are enrolling patients (NCT02274584 and NCT02414269); however, these residual cell populations and the possibility of iCasp9 dimerization independent of dimerizing agent potentially limit the widespread use of this strategy [93]. This selective depletion can also be mediated by the clinically approved therapeutic antibody when the transduced cells are engineered to express the antibody targeted cell surface antigen such as truncated EGFR (tEGFR) [94], a human EGFR polypeptide retaining the intact cetuximab binding site in extracellular domain III. Moreover, tEGFR can serve as a cell surface marker for the identification of the infused CAR-T cells in vivo and has been used in clinical trials [5, 9]. Nonetheless, whether this cell ablation through antibody-dependent cellular cytotoxicity can rapidly start in the event that severe toxicity occurs in humans remains undetermined and needs to be verified in forthcoming clinical trials.

## Conclusions

CAR-T cells are the best-in-class example of genetic engineering of T cells, bringing us spectacular opportunities and hopefully entering the mainstream of cancer therapy for B cell malignancies in the next 1–2 years. But tumor antigen escape relapse resulting from selective immune pressure of CAR-T cells highlights the shortcomings of this novel modality. Moreover, a similar surprise has not been elicited in the application of solid tumors with less efficacy and on-target/off-tumor toxicity, suggesting that enhancing the efficacy and safety of CAR-T cells should be considered as a starting point for the novel CAR design. Encouragingly, the proof-of-concept designs mentioned above to address these issues have been tested in experimental studies, providing preliminary evidence of feasibility and paving the road to further optimization. Of these designs, targeting more than one tumor antigen (i.e., dual-targeted CAR) should take the front seat due to it is not only beneficial to reducing or preventing the risk of antigen escape relapse either in hematological malignancies or solid tumors but also may alleviate the impact of antigenic heterogeneity on therapeutic effect in solid tumors. However, the prerequisite of the dual-targeted CAR for successfully offsetting antigen escape relapse is that it can effectively kill targets expressing either antigen, similarly to a monospecific CAR. This places a significant restriction on the implement in solid tumors as dual-targeted CAR potentially enhances the risk of on-target/off-tumor recognition compared to the unispecific CAR. In fact, as discussed above, the concept of using more than one target for CAR-T cell therapy in solid tumors mainly focuses on enhancing the specificity of CAR through the design of combinatorial antigen targeting, by which T cell only can be fully activated when the two target antigens are present at the same time. Above all, dual-targeted CAR is an optimal approach for overcoming antigen escape relapse with manageable on-target/off-tumor toxicity-B cell aplasia in B cell malignancies; however, it is still challenging to implement in solid tumors because it is difficult to balance the therapeutic effect and on-target/off-tumor toxicity. Combination tuning the sensitivity of CAR by scFv affinity with suicide gene may be a powerful strategy for broadening the application of dual-targeted CAR beyond hematological malignancies. However, the eventual effects of these novel designs still need to be determined in forthcoming clinical trials.

## Abbreviations

AFP: Alpha-fetoprotein
allo-SCT: Allogeneic stem cell transplantation
AML: Acute myeloid leukemia
B-ALL: B cell acute lymphoblastic leukemia
BiTE: Bispecific T engager
B-NHL: B cell non-Hodgkin’s lymphoma
CAR: Chimeric antigen receptor
CAR-T cells: Chimeric antigen receptor-modified T cells
CCR: Chimeric costimulatory receptor
CEA: Carcinoembryonic antigen
CLL: Chronic lymphocytic leukemia
CR: Complete remission
CSCs: Cancer stem cells
DLI: Donor lymphocyte infusion
EGFR: Epidermal growth factor receptor
EGFRvIII: Variant III of the epidermal growth factor receptor
FITC: Fluorescein isothiocyanate
GFP: Green fluorescent protein
GVHD: Graft-versus-host disease
GVT: Graft versus tumor
HER2: Human epidermal growth factor receptor-2
HL: Hodgkin’s lymphoma
HLA: Human leukocyte antigen
iCasp9: Inducible caspase-9
MHC: Major histocompatibility complex
MLL: Mixed lineage leukemia
MSKCC: Memorial Sloan Kettering Cancer Center
MSLN: Mesothelin
NSG: NOD/SCID/gamma-chain-deficient
OS: Overall survival
pMHC: Peptide-MHC
PMLBCL: Primary mediastinal large B cell lymphoma
PNE: Peptide neo-epitope
pre-BCR: Pre-B cell receptor
Pro-antibody: Protease-activated antibody
PSCA: Prostate stem cell antigen
PSMA: Prostate-specific membrane antigen
scFv: Single-chain variable fragment
SynNotch: Synthetic Notch receptors
TAA: Tumor associate antigen
TCR: T cell receptor
tEGFR: Truncated EGFR
Upenn: University of Pennsylvania
